# Preclinical Evidence of Rapid-Onset Antidepressant-Like Effect in Radix Polygalae Extract

**DOI:** 10.1371/journal.pone.0088617

**Published:** 2014-02-10

**Authors:** Im-Joon Shin, Sung Un Son, Hyunwoo Park, Yoorim Kim, Sung Hyun Park, Kelley Swanberg, Joo-Yeon Shin, Sang-Kyu Ha, Yoonju Cho, Soo-Yong Bang, Jae-Hwan Lew, Seung-Hun Cho, Sungho Maeng

**Affiliations:** 1 Department of Applied Korean Medicine, College of Oriental Medicine, Kyung Hee University, Seoul, Korea; 2 Department of East-West Medicine, Graduate School of East-West Medical Science, Kyung Hee University, Yongin, Korea; 3 Department of Neuropsychiatry, College of Oriental Medicine, Kyung Hee University, Seoul, Korea; Chiba University Center for Forensic Mental Health, Japan

## Abstract

Radix Polygalae (the root of *Polygala tenuifolia*) is a herb widely used in traditional Asian medicine that is thought to exert a variety of neuropsychiatric effects. Radix Polygalae extract can protect against N-methyl D-aspartate (NMDA) neurotoxicity and induce brain-derived neurotrophic factor (BDNF) expression, suggesting modulatory roles at glutamatergic synapses and possible antidepressant action. In accordance with this hypothesis, Radix Polygalae extract demonstrated antidepressant-like effects in 8-week-old male C57Bl/6 mice by decreasing behavioral despair in the forced swim and tail suspension tasks and increasing hedonic-like behavior in the female urine sniffing test 30 minutes after a single oral administration of 0.1 mg/kg. Reduced latency to acquire a food pellet in the novely suppressed feeding paradigm, without change in anxiety-like behaviors suggested a rapid-onset nature of the antidepressant-like effect. In addition, it decreased the number of failed escapes in the learned helplessness paradigm after two oral administrations 24 hours and 30 minutes before the first test. Finally, it reversed anhedonia as measured by saccharin preference in mice exposed to the chronic stress model after two administrations of 0.1 mg/kg, in contrast to the repeated administration generally needed for similar effect by monoamergic antidepressants. Immobility reduction in tail suspension task was blocked by the α-amino-3-hydroxy-5-methyl-4-isoxazolepropionic acid (AMPA) receptor antagonist NBQX, a pattern previously demonstrated by ketamine and other ketamine-like rapid-onset antidepressants. Also similarly to ketamine, Radix Polygalae appeared to acutely decrease phosphorylation of GluR1 serine-845 in the hippocampus while leaving the phosphorylation of hippocampal mTOR serine 2448 unchanged. These findings serve as preclinical evidence that Radix Polygalae extract exerts rapid-onset antidepressant effects by modulating glutamatergic synapses in critical brain circuits of depression and may be worthy of further evaluation as a safe substitute to other rapid-onset antidepressants known to have unacceptable side effects.

## Introduction

Depressive disorder was reported as the second leading cause of disability in the 2010 Global Burden of Disease Study, accounting for over an estimated 60 million years lived with disability across the world population [1]. Although patients of depression have a wide variety of pharmacological agents with which to attempt treatment, multiple clinical studies have called into question the effectiveness of antidepressants in preventing and ameliorating depressive episodes [2]. Individuals undergoing antidepressant therapy can experience high rates of relapse, persistent residual symptoms, and intolerable side effects [3]–[5]. Moreover, a major limitation of antidepressants is their lag period for therapeutic effects, which has led to growing interest in rapid-onset pharmacological alternatives [6].

The glutamate hypothesis of depression arose in the early 1990s when it was found that the glutamate receptors in the frontal cortices of suicide victims bound glycine at a lower rate than those of controls [7]. A concomitant flurry of preclinical and clinical trials suggested that drugs affecting the glutamate system, including D-cycloserine (partial NMDA agonist) [8], amantadine (NMDA antagonist) [9], lamotrigine (glutamate release inhibitor) [10], and riluzole (glutamate release inhibitor and AMPA potentiator) [11] may have antidepressive properties. Research began to indicate that unlike the majority of commonly prescribed antidepressants, pharmaceuticals putatively targeting glutamatergic signaling pathways had the capacity to exert therapeutic effect within acute time frames. As a poignant example, a study by Berman et al. [12] showed that NMDA antagonist ketamine improved the mean Hamilton depression rating scale (HDRS) score within 4 hours in patients not responding to conventional antidepressants. Following this, a study by Zarate et al. [13] demonstrated antidepressant effects for at least a week after a single administration of ketamine in a double-blind, placebo-controlled randomized crossover trial. Furthermore, in a study on mice, preoccupation of AMPA receptors blocked the mood-improving effect of ketamine, suggesting that the change of AMPA-to-NMDA throughput is key to the emergence of this NMDA antagonist's antidepressant effect [14]. In accordance to the antidepressant mechanism of NMDA antagonists, change of GluR1 and mTOR phosphorylation in the glutamatergic synpases were suggested as critical molecular mechanisms associated with rapid-onset antidepressant-like effects [14], [15]. But as ketamine is not appropriate for clinical use due to its untoward effects and potential for abuse [16], a search for substitute reagents is in progress.

Radix Polygalae (RP) is the dried root of *Polygala tenuifolia*, which has been traditionally used across several East Asian cultures as an expectorant, tranquillizer, and anti-amnesia reagent. It has been found to contain several varieties of saponins and xanthones that are the putative mediators of an array of biological activities, including D_2_ and 5-HT_2_ receptor binding in rat striatal and cortical tissues, respectively, and inhibition of norephinephrine transporter protein in transfected canine kidney cells [17]–[19]. RP extract has reduced dopamine-induced oxidative stress and protected cortical neurons from NMDA toxicity [20], [21]. YZ-50, one of the fractioned components of RP, showed antidepressant-like effects in the FST and TST as well as on the sucrose consumption of chronically mild stressed (CMS) animals [22], [23]. YZ-50 also increased brain-derived neurotrophic factor (BDNF) and Tyrosine kinase B (TrkB) receptor expression, which may induce hippocampal neurogenesis similarly to other antidepressant reagents [24]. Because RP protected cultured neurons from NMDA toxicity and induced BDNF expression [25], we hypothesized that it modulates glutamatergic synapses to effect rapid-onset (within minutes or hours) antidepressant-like properties.

This hypothesis was tested in the present study using behavioral methods coupled with examination of the biochemical changes of brain tissue in regions previously implicated in depression. To determine whether Radix Polygalae reduced immobility in mouse models of acute behavioral despair, a popular method of screening for putative antidepressants, we tested one set of mice on the tail suspension (TST) and forced swim tests (FST) after various doses and time scales of oral RP administration, including after pretreatment with AMPA antagonist NBQX. To confirm RP's acute-onset antidepressant-like effect in models of hedonic behavior, novelty-driven stress, and learned helplessness, we then tested two more groups of mice on the female urine sniffing test (FUST) and novelty-suppressed feeding test (NSFT) as well as on the learned helplessness (LH) battery, respectively, following acute RP administration on separate days. In order to bolster these acute analyses with evidence of RP's antidepressant action in a depression model of greater face, construct, and predictive validity than the acute behavioral measures used for screening, we then determined its effect on body mass, saccharin preference, and FST immobility in a fourth group of mice subjected to a six-week chronic mild stress (CMS) model. Finally, to determine whether RP, similarly to previously described NMDA antagonists with rapid-onset antidepressant effect, affects the phosphorylation of NMDA subunit GluR and mTOR in the hippocampus, an immunoblotting assay was carried out on hippocampal brain tissue extracted from a final cohort of mice following acute RP administration.

## Methods

### Animals

Eight-week old male C57Bl/6 and ICR mice were purchased from Samtaco (Suwon, Korea). These mice were housed in temperature- and humidity-controlled conditions with 12-hour light-dark cycles and were allowed access to rodent chow and water ad libitum until the start of the behavioral experiments. Except for the learned helplessness paradigm (using ICR mice), every other behavioral experiment was performed using C57Bl/6 mice.

### Ethics Statement

The animal studies were conducted in accordance with the Guide for the Care and Use of Laboratory Animals as adopted and promulgated by the National Institutes of Health, and all protocols were approved by the Institutional Animal Care and Use Community of Kyung Hee University (KHMC-IACUC: 10-071).

### Plant material

Radix Polygalae was purchased from Kyung Hee University Medical Center (Seoul, Korea) and authenticated by Professor Jae-Hwan Lew. A voucher specimen was deposited in the Kyung Hee University College of Oriental Medicine herbarium.

### Preparation of Radix Polygalae extract

The dried RP samples were immersed in 70% ethanol and boiled at 80°C for 1 hour. The extract was collected and the procedure repeated once more (80°C reflux for 40 min), after which the combined filtrate was evaporated on a rotary evaporator under reduced pressure and freeze-dried to yield about 28% (w/w) of the extract. Supporting information is available for the HPLC quantification of key molecules (Figure S1). For experimental use, crude extracts were completely dissolved in distilled water.

### Behaviors

Tail suspension test (TST): To screen for antidepressant-like effects in RP, TST was carried out according to the method described by Steru et al. [26] with minor modifications. To screen for dose dependency, RP was administered orally 30 minutes prior to testing at doses of 0.1 mg/kg, 1 mg/kg, and 10 mg/kg. To screen for time dependency, RP (0.1 mg/kg) was given orally 30, 60, and 120 minutes before the test. The control group was administered a comparable volume of distilled water 30 minutes before the test.

The involvement of AMPA receptors in the antidepressant-like effect of RP in the TST was checked with the use of AMPA receptor antagonist NBQX. Immobility in the TST was determined after co-treatment of 10 mg/kg NBQX (i.p., 40 min before test) and 0.1 mg/kg RP (p.o., 30 minutes before test). The control group was injected with normal saline (40 min before test) and distilled water (30 minutes before test). Mice were suspended by the tail from a metal rod using adhesive tape and their movements videotaped for 6 minutes. Mobility time during the 6 minute test session was later determined by blind review of video files and subtracted from total test time to determine immobility. Mobility was defined as movement of the hind legs. Any mice that climbed their tails during the test were excluded from data analysis.

Forced swim test (FST): Another screen for acute antidepressant-like effect, FST was carried out as reported in Borsini and Meli [27]. RP was administered orally 30 minutes prior to testing at doses of 0.1 mg/kg, 1 mg/kg, and 10 mg/kg. The control group was administered a comparable volume of distilled water. Each mouse was placed into a transparent Plexiglas cylinder (height 35 cm, diameter 20 cm) filled with tap water (23–25°C) to 21.5 cm and their movements videotaped. Immobility time during the last 4 minutes of the 6 minute test session was later determined by blind review of video files. Immobility was defined as the minimal movement required to maintain the head above the water.

Female urine sniffing test (FUST): Reward-seeking activity was measured by the female urine sniffing test, adapted from Malkesman et al. [28]. The FUST is based on the sexual drives of male mice to smell the urine of females in estrus [28]. Briefly, mice were habituated to the presence of a sterile cotton-tipped applicator placed into the home cage for an hour prior to testing. After habituation, each mouse was separately transferred to a quiet room with low (<20 lux) ambient lighting and exposed for 3 minutes to a cotton tip dipped in distilled water. After a 45 minute interval, the mouse was exposed to a cotton tip infused with fresh urine collected from females of the same strain in estrus. Duration of sniffing was measured during exposure to both water and urine. RP extract (0.1 mg/kg) or comparable volumes of distilled water were administered orally 30 minutes before the exposure to both distilled water and female urine.

Novelty suppressed feeding test (NSFT): The ease with which feeding behavior was suppressed by the stress of a novel environment, a trait shown to decrease after chronic but not subchronic or acute antidepressant treatment [29], was measured to further evaluate the manner of antidepressant effect in RP. NSFT was conducted as described previously with minor modifications [30]. Briefly, for 24 hours before the test the mice were deprived of food but not water. On the day of the test, mice were placed at the edge of the Plexiglass test chamber (60 cm×60 cm) with a single food pellet in the center in a quiet room with dim lighting (<20 lux). Latency to feeding was measured for 5 minutes; non-feeding behaviors (e.g, touching, smelling) were ignored. If food was not taken within 5 minutes, feeding latency was regarded as 5 minutes. The mice were then returned to their home cages, and latency to feeding was measured again. RP (0.1 mg/kg) or a comparable volume of distilled water was orally administered 30 minutes before the test.

Learned helplessness (LH) paradigm: Learned helplessness, or the loss of escape behaviors from an aversive situation based on the repeated futility of previous attempts to escape, is considered relevant to the symptomology of both depression in humans and models thereof in rodents [31]. Learned helplessness was induced and its response to RP assessed with the Gemini Avoidance system (San Diego Instruments, San Diego, California) according to the protocol described in [14]. Briefly, LH testing was conducted over the course of four days and divided into induction, screening, and testing phases. Helplessness was induced by placing mice into one closed chamber of the Gemini 2-way avoidance shuttle box and allowing them to habituate for 300 seconds before exposing them to a conditioned stimulus (light and white noise) for 1 second, following which the conditioned stimulus was repeated while an inescapable 0.45-mA electric shock through the floor was delivered for 15 seconds. 120 trials were conducted each day for two days; intertrial intervals ranged from 4 to 48 seconds (average 26 seconds). On the third day, screening for numbers of escape failures per mouse was performed by placing each mouse into the now-open shuttle box chamber for 300 seconds of habituation, followed by with 3 seconds of conditioned stimulus exposure and 3 seconds of conditioned stimulus plus 0.45-mA foot shock. Each mouse underwent 30 screening sessions with an inter-trial interval of 12 to 38 seconds (average 25 seconds). Only mice that developed helplessness (more than 20 escape failures) were used for further evaluation. Numbers of escape failures were measured the next day in this newly vetted group after two administrations of either RP extract (0.1 mg/kg, p.o.), fluoxetine (20 mg/kg, i.p.), or distilled water given 24 hours and 30 minutes before the test, which was identical to screening except that shocks were conducted for 24 instead of 3 seconds (30 trials; 0.45 mA, 12 to 38 seconds intertrial intervals, average of 25 seconds). After an additional 8 days, a second test for escape failures was conducted without any further treatment.

Elevated plus maze (EPM): The effect of RP on anxiety-like behavior was measured by the Elevated Plus Maze, a test of risk-taking behavior that has demonstrated sensitivity to acute treatment with anxiolytics [32]. Our maze device was a white opaque Plexiglass maze of four perpendicular 5×27-cm arms extending lengthwise out from the 5 cm×5 cm center and elevated on stilts to 40 cm above the ground. Two opposite arms were open, while two were enclosed by a pair of opaque 16-cm walls. The mouse was placed in the center region of the maze with its head pointing toward an open arm. The movement of the mouse through the maze arms was recorded for 5 minutes, and the time spent in open arms was calculated using video-tracking software (Smart 3.0, Panlab, Spain). RP (0.1 mg/kg) was orally administered 30 minutes before the test.

Chronic mild stress (CMS): Mild unpredictable stressors applied over several weeks have been shown to elicit anhedonic and other depression-like phenotypes in mice, leading to the acceptance of various chronic stress protocols as satisfactorily valid depression models in rodents [33]. The particular CMS regimen used in this study was adapted from a previously described protocol [34]. Briefly, the CMS procedure was applied to individually housed mice for six weeks. It consisted of a variety of stressors, including 24 hours of food and water deprivation, stroboscope illumination, white noise, overnight illumination, 45° home cage tilt, wet bedding, exposure to novel objects in the home cage, and confinement to a small tube (Table 1), applied in sets of two to three over any 24 hour period in a pseudo-random order throughout the experiment. Control animals were individually housed for the six-week period without the delivery of extra stressors.

**Table 1:** Procedure of chronic mild stress.

Week	Mon	Tue	Wed	Thu	Fri	Sat	Sun
	WN (2 h)	CF (1 h)	SI (3 h)	CF (1 h)	SI (3 h)		SPT
1	SI (3 h)	WN (2 h)	CF (1 h)	WN (2 h)	CF (1 h)	FWD (18 h)	NO (18 h)
	CF (1 h)	LO (12 h)	WB (12 h)	TC (12 h)	TC (12 h)		
	CF (1 h)	SI (3 h)	WN (2 h)	SI (3 h)	WN (2 h)		SPT
2	SI (3 h)	CF (1 h)	SI (3 h)	WN (2 h)	LO (12 h)	FWD (18 h)	NO (18 h)
	WN (2 h)	TC (12 h)	WB (12 h)	CF (1 h)			
	WN (2 h)	CF (1 h)	WN (2 h)	SI (3 h)	SI (3 h)		SPT
3	CF (1 h)	SI (3 h)	CF (1 h)	TC (12 h)	CF (1 h)	FWD (18 h)	NO (18 h)
	LO (12 h)	WN (2 h)	WB (12 h)		WN (2 h)		
	SI (3 h)	SI (3 h)	CF (1 h)	WN (2 h)	WN (2 h)		SPT
4	WN (2 h)	CF (1 h)	SI (3 h)	SI (3 h)	CF (1 h)	FWD (18 h)	NO (18 h)
	CF (1 h)	TC (12 h)	WB (12 h)		TC (12 h)		
	CF (1 h)	SI (3 h)	CF (1 h)	SI (3 h)	WN (2 h)		SPT
5	WN (2 h)	WN (2 h)	WN (2 h)	CF (1 h)	SI (3 h)	FWD (18 h)	NO (18 h)
	LO (12 h)		WB (12 h)	TC (12 h)	CF (1 h)		
	WN (2 h)	SI (3 h)	WN (2 h)	CF (1 h)	WN (2 h)		
6	CF (1 h)	CF (1 h)	CF (1 h)	WN (2 h)	SI (3 h)	FWD (18 h)	SPT
	TC (12 h)	LO (12 h)	WB (12 h)		CF (1 h)		

CF: confinement, FWD: food and water deprivation, LO: light on, NO: novelty object, SI: stroboscope illumination, SPT: saccharin preference test, TC : tilting cage, WB : wet bedding, WN : white noise (80dB).

The consequences of the CMS were evaluated through body mass, saccharin preference as a commonly applied proxy for anhedonic behavior, and FST immobility. Body weight was measured weekly, following which mice were deprived of food and water for 18 hours. Saccharin (0.1%) consumption was then measured with a two-bottle protocol by which mice were exposed to a bottle each of distilled water and 0.1% saccharin solution for 18 more hours. Saccharin and water consumption were calculated as the difference in bottle masses before and after the test; saccharin preference (%) was the ratio of saccharin consumption to saccharin plus water consumption.

RP (0.1 mg/kg; p.o.), ketamine (10 mg/kg; i.p.), or distilled water (p.o.) was administered 24 h and 30 minutes before the saccharin consumption measurement in the sixth week of CMS exposure. The next day, FST was performed after an additional dose of RP or ketamine was given 30 minutes before testing according to the methods described previously.

### Immunoblotting

The effect of RP on GluR1 and mTOR phosphorylation was measured by immunoblotting. 30 minutes after a single oral administration of RP (0.1 mg/kg), mice were sacrificed through anesthesia overdose, and their brains were extracted. The hippocampus was isolated and kept in a −70°C deep freezer until use. In order to dissolve hippocampal tissue for denaturing, 300 µL of lysis buffer (20 mM Tris HCI, 150 mM NaCl, 1 mM EDTA, 1 mM EGTA, 1% TritonX-100, 2.5 mM sodium pyrophosphate, 1 mM EGTA, 1% TritonX-100, 2.5 mM sodiumpyrophosphate, 1 mM beta-glycerophosphate, and freshly added 1% protease inhibitor, 1% phosphatase inhibitor I, 1% phosphatase inhibitor II) was added and the tissue homogenized in a tissue grinder (Krackeler Scientific, Inc. Albany, NY). After the concentration of protein was measured to 2 mg/mL in accordance with the Bradford method, homogenates were boiled for 4 minutes to denature the proteins. Samples for western blots were resolved on polyacrylamide gel using an electrical current in accordance with the SDS-PAGE method. After that, the separated proteins were transferred to PVDF membrane. After the protein resolution was checked with Ponseau S solution, the membrane was washed for 5 minutes with TBS-T (Tris buffered saline +0.5% tween-20) and blocked for 1 hour by immersing it in 5% skim milk dissolved in TBS-T. Primary antibodies (anti-gluR1 serine 845, anti-gluR1, anti-mTOR serine 2448, anti-mTOR; Cell Signaling Tech. Inc., Danvers, MA) were diluted with a mixture of 2.5% skim milk and 2.5% BSA (bovine serum albumin) in a 1∶1000 concentration ratio and reacted with the membrane at 4°C for one night. On the next day, after 3 washings in TBS-T, HRP (horseradish peroxidase)-conjugated secondary antibody was added to detect the primary antibody. The secondary antibody was diluted with 5% skim milk in a 1∶1000 concentration ratio. After the membrane was incubated with the secondary antibody, it was washed with TBS-T solution for 5 minutes three times in a shaker. Then ECL solution was applied to the membrane for 5 minutes, and a protein band was made by using a chemiluminiscence detector (Davinch-Chemi imaging system, CellTagen, Korea). Quantitative analysis was performed on band density with ImageJ 1.46 r (NIH, USA) image analyzing software. For reprobing, stripping buffer (2% SDS, 100 mM beta-mercaptoethanol, 50 mM Tris, pH 6.8) was applied to membranes, which were then incubated for 20 minutes in a water bath heated to 50° C.

### Statistics

SPSS 20 (IBM Software) was used for statistical analysis. One and two-way ANOVA and t-tests were applied to between-group comparisons; Tukey's Honestly Significant Differences test was performed following confirmation of significant ANOVA results. The threshold for statistical significance was set at p<0.05. All results are indicated as the mean ± standard error of the mean (SEM).

## Results and Discussion

### Screening for dose and time dependency of Radix Polygalae's antidepressant effect

**Figure 1:**
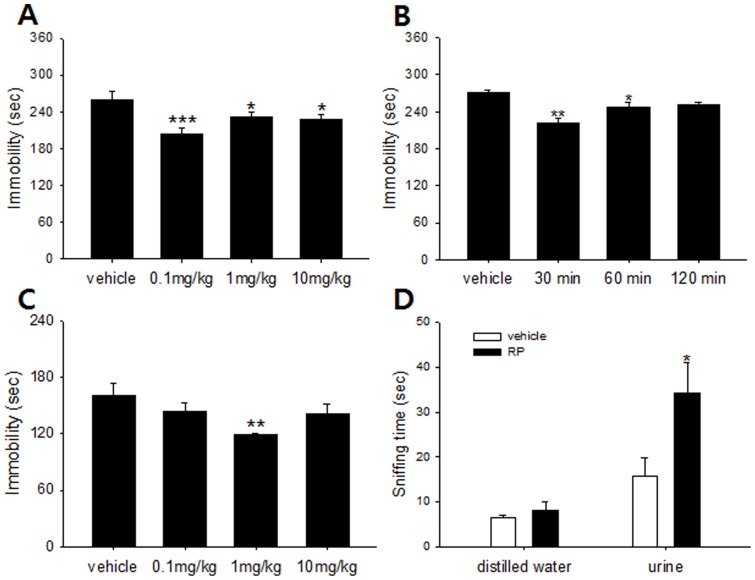
Antidepressant-like effect of Radix Polygalae in mouse models of behavioral despair and hedonism. (A) Immobility in tail suspension test measured 30 minutes after RP (0.1, 1 and 10 mg/kg p.o.). There was a significant dose effect [F(3,31) = 5.62, p = 0.0038]. Immobility reduction was most prominent in the 0.1 mg/kg group (p<0.001). (B) Immobility in tail suspension test measured 30, 60, and 120 minutes after 0.1 mg/kg RP administration. There was a significant difference among groups [F(3,31) = 11.4, p<0.001]. RP administered 30 minutes before the test induced the most significant reduction in immobility (p = 0.005). (C) Immobility in forced swim test measured 30 minutes after RP (0.1, 1, and 10 mg/kg p.o.). A significant difference among doses emerged [F(3,31) = 2.99, p = 0.048]. RP 1 mg/kg reduced immobility relative to distilled water controls (p = 0.006). (D) Sniffing time in female urine sniffing test. No differences between mice administered RP (0.1 mg/kg p.o.) and distilled water controls emerged in the sniffing time of distilled water, but sniffing time to estrus female urine significantly increased in the RP group relative to control (p = 0.02). Vehicle: distilled water, RP: Radix Polygalae extract. All data represent mean ± SEM. * p<0.05, ** p<0.01 *** p<0.001 vs controls. ANOVA, Tukey's post hoc test for (A), (B) and (C). Student t-test for (D).

The first series of experiments demonstrated an antidepressant-like effect of RP in two mouse models of behavioral despair. In the TST (n = 8 per group), RP had a significant dose effect, and significant decreases in immobility were seen at 0.1 mg/kg (p<0.001), 1 mg/kg (p = 0.047), and 10 mg/kg (p = 0.027) relative to control; it was most prominent at a 0.1 mg/kg oral dose (Figure 1A). The FST (n = 8 per group) also exhibited a significant dose effect, but 1 mg/kg was the most effective dose for reducing immobility relative to control (Figure 1C). It is not clear why the maximal effective dose was different between these two screens, but it is believed that the biological substrates mediating performance in the FST and TST are not identical, and large differences in baseline immobility between the two tasks can exist [35]. The TST has shown sensitivity to the effects of some anxiolytics, manifesting as increased immobility following acute administration [36]. While our open field and EPM results suggested the absence of anxiolytic and sedative effects for 0.1 mg/kg RP (see below), 1 mg/kg was not tested for these influences, so it is possible that TST, but not FST, immobility scores at this dose were artificially increased by this effect. However, the potential confound of RP's possible anxiolytic effect at higher doses does not explain why the 0.1 mg/kg dose effected significant immobility decreases in the TST but not the FST; it is thus likely that other inter-test differences beyond their relative sensitivities to anti-stress influences may also have played a role in this disparity.

Indeed, at a higher dose (100 mg/kg) of RP, mice became lethargic and fell asleep, probably due to sedation (data not shown). This pattern is similar to that shown by ketamine, an NMDA antagonist that has demonstrated rapid-onset and sustained antidepressant effect in a number of other studies. Ketamine has also shown to have rapid-onset antidepressant effect only at sub-anesthetic doses, while higher doses induce dissociative anesthesia [12], [37]. Although there were differences between the most effective doses in the FST and TST, the antidepressant-like effects of RP were exerted by lower doses that may not induce sedation. This is an interesting aspect shared by some rapid-onset antidepressants [13], [38].

Immobility reduction in TST and FST needs to be differentiated from psychomotor activation, which can lead to false impressions of an antidepressant-like effect. This was tested in the open field, by measuring the locomotor activity after a single dose of 0.1 mg/kg RP (n = 6 per group) (Figure S2A). Compared with vehicle (distilled water) treated mice, there were no significant differences from RP-treated mice.

As a result of its greater sensitivity to a wider range of doses, the TST was used as the source for our baseline 0.1 mg/kg as the dose applied in further tests. As separate screening for drug incubation time in the TST showed a significant effect of dose timing (n = 8 per group) and that, in particular, a 30-minute treatment-test interval was the most effective (Figure 1B), RP administration was performed 30 minutes before all further tests.

Next, we used the female urine sniffing test (FUST) to examine whether RP affects reward-seeking behaviors (n = 6 per group). There was no difference between control and 0.1 mg/kg RP-treated mice in time spent sniffing distilled water, but RP-treated mice spent more time than control mice sniffing estrus female urine (Figure 1D), suggesting that RP administration increased their propensity for reward seeking. As anhedonia is one of the symptomatic hallmarks of depression [39], the increase in reward seeking following RP administration may serve as evidence of the substance's antidepressant-like influences on brain function.

### Assessing the rapid-onset antidepressant-like effect of Radix Polygalae

Following screening for dose and time dependency, RP's rapid antidepressant-like effect was assessed with the Novelty Suppressed Feeding Test (NSFT), and learned helplessness (LH) paradigm. Additional assessments of saccharin preference and FST immobility were also made following six weeks of chronic stress.

**Figure 2:**
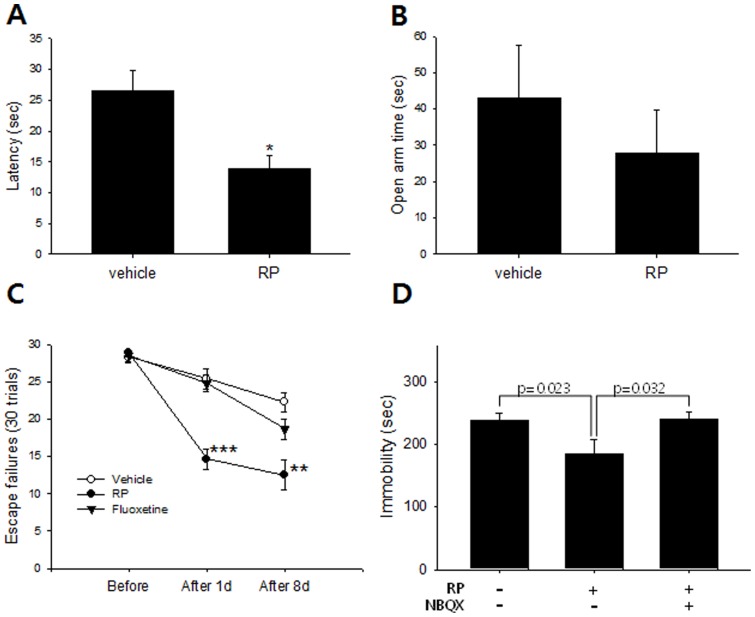
Acute-onset antidepressant-like effect of Radix Polygalae in mice. (A) Novelty suppressed feeding test. Latency to take a food pellet was reduced by RP (0.1 mg/kg, p.o.) administered 30 minutes before test, suggesting either rapid-onset anxiolytic or antidepressant-like effect. * p<0.05, student t-test. (B) Elevated plus maze. There was no statistical difference between mice administered RP (0.1 mg/kg, p.o.) or controls administered distilled water in the total time spent in open arms which meant RP does not change anxiety-like behaviors at the given dose. (C) Learned helplessness paradigm. Number of escape failures reduced within 24 hours after RP treatment (0.1 mg/kg, p.o.) but not by fluoxetine (20 mg/kg i.p.), suggesting a rapid-onset antidepressant-like effect. ** p<0.01 *** p<0.001 vs vehicle on the same day. Two-way ANOVA, Tukey's post hoc test. (D) Tail suspension test. The antidepressant-like effect of RP (0.1 mg/kg, p.o.) was blocked by NBQX injection (10 mg/kg). ANOVA, Tukey's post hoc test. Vehicle: distilled water, RP: Radix Polygalae. All data represent mean ± SEM.

In the novelty suppressed feeding test (NSFT), both acutely administered anxiolytics and chronic antidepressants contribute to reduced feeding latency; usually at least 3 weeks of the latter is required for a significant effect [29]. But a single oral administration of RP (0.1 mg/kg, 30 minutes prior to testing) reduced the feeding latency from control levels to a significantly lower treatment level in NSFT (n = 8 per group; Figure 2A). To confirm that this between-group difference was attributable to antidepressant rather than anxiolytic effect, the same dose (0.1 mg/kg, 30 minutes before test) was assessed on the Elevated Plus Maze (n = 8 per group; Figure 2B). Time spent in the open arms did not increase in the RP-treated group relative to control, suggesting that RP treatment did not induce any change in anxiety-like behaviors at the given dose and thus decreasing the probability that the between-group differences noted in the NFST were due to anxiolytic effect. Finally, as appetite changes may also affect NSFT latency, the effect of RP on the amount of chow consumption was measured. There was no significant difference in food pellet consumption over 24 hours following a single oral administration of either 0.1 mg/kg RP or distilled water (n = 6 per group; Figure S2B).

The learned helplessness (LH) paradigm is a pharmacological model of depression that responds to repeated administration of antidepressants [40]. After a series of repetitive and inescapable foot shocks induces behavioral despair, the number of failures to avoid shock is counted while the animals are permitted to escape the shock chamber before receiving a shock. The number of escape failures showed a significant effect of treatment (n = 8 per group), showing a reduction in escape failures maintained from the first to the eighth days from screening following two doses of RP (0.1 mg/kg p.o.) 24 hours and 30 minutes before the first test compared to vehicle- and fluoxetine-treated mice (Figure 2C). Meanwhile, the number of escape failures was not reduced in mice treated with two injections of fluoxetine (20 mg/kg) administered 24 hours and 30 minutes before the first test.

**Figure 3:**
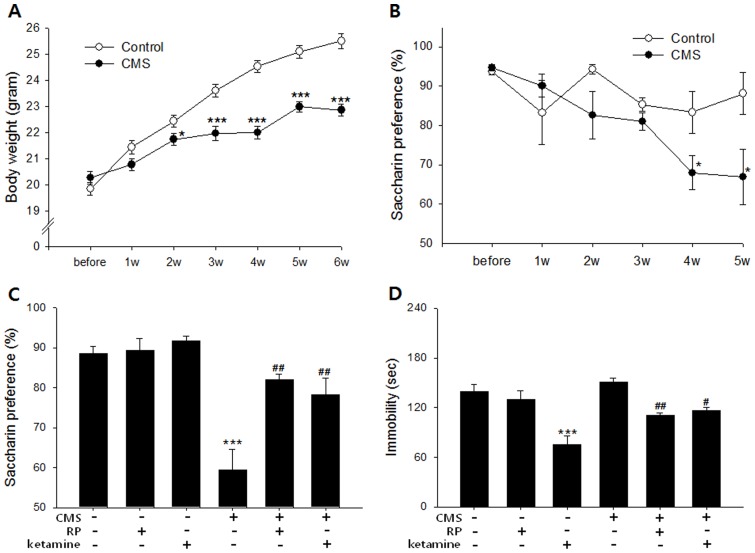
Antidepressant-like effect of Radix Polygalae in mouse models of chronic stress. CMS was delivered to mice for 6 weeks as described in methods. (A) Body weight. CMS mice gained less body weight compared with unstressed controls [repeated measures ANOVA; F = 136.0, p<0.001]. (B) Saccharin preference. CMS mice showed a gradual decrease of preference [repeated measures ANOVA; F = 12.3, p = 0.001]. (C) Saccharin preference after treatment of either vehicle (normal saline), RP (0.1 mg/kg p.o.), or ketamine (10 mg/kg i.p.) administered 24 hours and 30 minutes before measurement to mice exposed to CMS for 6 weeks. A significant difference existed between treatment groups [F(5,29) = 18.1, p<0.001]. Among CMS exposed mice, RP (p = 0.002) and ketamine (p = 0.005)-treated mice showed a significantly higher preference than vehicle treated mice. (D) Immobility in forced swim test from mice exposed to CMS for 6 weeks. Mice were given either vehicle (normal saline), RP (0.1 mg/kg p.o.), or ketamine (10 mg/kg i.p.) administered 48 hours, 24 hours, and 30 minutes before test. A significant group difference emerged [F(5,29) = 12.9, p<0.001)]. In CMS exposed mice, RP (p = 0.004), and ketamine (p = 0.02) reduced immobility. CMS: chronic mild stress. RP: Radix Polygalae. All data represent mean ± SEM. * p<0.05, ** p<0.01 *** p<0.001 vs control or vehicle-treated non-CMS. ^#^ p<0.05, ^##^ p<0.01, vs vehicle-treated CMS. 2-way ANOVA for (A) and (B), ANOVA for (C) and (D). Tukey's post hoc test.

For further validation that the behavioral changes associated with the RP-treated groups in the FST, TST, FUST, NSFT, and learned helplessness paradigms may be ascribed to antidepressant effect, RP's influence on saccharin preference and FST immobility were measured in mice exposed to a chronic stress paradigm. As a model of depression, CMS has enhanced predictive, face, and construct validity relative to one-off screens of acutely stressed animals and has been shown to be most sensitive to antidepressant administration over chronic time scales [33], though one recent study also demonstrated reversal of post-CMS sucrose preference losses after just one injection of ketamine [41]. The saccharin preference test, insofar as it measures reductions in a mouse's putative natural preference for sweet drink over plain water, is, like the FUST, a commonly used battery of anhedonia, a symptom commonly associated with both rodents exposed to the CMS and depressive patients [42]. It avoids the confounding influence of metabolic differences on ingestion over time by using non-caloric saccharin instead of high-calorie sucrose, though both variants have appeared in the literature. During CMS exposure, both weight gain and saccharin preference of the stressed animals was significantly reduced compared with controls (n = 5 per group; Figure 3A & 3B). At 6 weeks, the saccharin preferences of CMS animals treated with two oral administrations of RP (0.1 mg/kg p.o.; 24 hours and 30 minutes before test) or ketamine (10 mg/kg, 24 hours and 30 minutes before test) increased significantly compared with those of vehicle-treated animals (distilled water; Figure 3C). This finding was comparable to that of a previous study in which rats administered YZ-50, a fraction of RP ethanol extract, for 28 days demonstrated increased sucrose consumption relative to CMS animals [22]. Our results, however, indicated a rapid as opposed to chronic onset of antidepressant-like effect because just two treatments of RP were sufficient to induce preference increases relative to control. Ketamine, which is well known for rapid-onset antidepressant effects in depressed patients, also induced a rapid change in hedonic behavior as measured by saccharin preference as well as behavioral despair as measured by FST immobility (Figures 3C & 3D). Post-CMS FST immobility also showed a significiant effect of treatment (n = 5 per group) in that the immobility of mice treated with three administrations of RP (0.1 mg/kg; 48 hours, 24 hours and 30 minutes before test) or ketamine (10 mg/kg; 48 hours, 24 hours and 30 minutes before test) was significantly reduced compared with that of vehicle treated animals (Figure 3D).

It was concluded from these data that RP exerts rapid-onset antidepressant-like effects in mice. This conclusion is supported by our findings that RP can acutely 1) reduce immobility in the FST and TST without demonstrating psychomotor effects in the open-field test, 2) reduce the feeding latency in NSFT without showing anxiolytic effects in the EPM, 3) reduce the number of escape failures in the LH paradigm, and 4) increase saccharin preference and reduce FST immobility in chronically stressed mice.

These findings support previous reports suggesting that RP contains multiple compounds that can exert antidepressant-like effects in rodents. YZ-50, a fraction of RP, reduced FST and TST immobility times in the FST and TST [23] and, as mentioned, increased sucrose preference in CMS rats [22]. Furthermore, 3,6′-disinapoyl sucrose (DISS), an active oligosaccharide ester component from RP, increased sucrose consumption in CMS-treated rats [43]. While the increase in hippocampal brain-derived neurotrophic factor (BDNF) expression and hypothalamo-pituitary-adrenal (HPA) axis suppression over a chronic time scale were suggested as underlying mechanisms for the antidepressant-like properties of these active ingredients [22], [43], [44], these compounds were administered multiple times over several weeks, allowing time for the development of such long-term effects that our study did not provide. Moreover, they were conducted on single compounds or fractions of RP extract that were potentially omitting the active ingredient(s) inducing rapid-onset antidepressant-like effects in ours. Indeed, other ingredients of RP have provided evidence of slightly different benefits. As an example, tenuigenin orally administered for seven days after ischemia-reperfusion injury in rats effected performance enhancements in the Y-maze and step-down tasks that were traced to increased levels of NMDA receptor 2B mRNA [45]. Finally, it is certainly possible that a number of putative mechanisms for rapid-onset antidepressant-like effect and long-term changes in BDNF production and HPA axis activation effected by one or more RP ingedients are not mutually exclusive and might even be casually linked, but such a hypothesis would need to be the subject of a longitudinal study on the acute and chronic effect mechanisms of single compounds within the extract.

Because in our study RP demonstrated acute-onset antidepressant-like effects, we tested the extent to which AMPA-dependent changes in glutamatergic (putatively NMDA) transmission, previously shown to be affected by rapid-onset antidepressants like ketamine [14] and other rapid-onset antidepressant compounds like the partial NMDA agonist GLYX-13 [46], might contribute to its therapeutic actions. RP was tested by its co-administration with NBQX, an AMPA receptor antagonist. In our findings, the immobility-reducing effect of 0.1 mg/kg RP in the TST was abolished by pretreatment with 10 mg/kg NBQX (p = 0.024; Figure 2D). NBQX has been shown to abolish the antidepressant effect of NMDA antagonists, suggesting that the change of NMDA to AMPA throughput in glutamatergic synapse is a key mechanism of their pharmacological effects [14]. It has abolished the antidepressant-like effects of Ro 25-6981 (NMDA receptor NR2B specific antagonist), MGS0039 (mGluRII antagonist), and CX546 (AMPAkine), all of which demonstrated rapid-onset antidepressant activities through modulation of the glutamate system [14], [47]. NBQX does not block, however, the pharmacological effect of monoaminergic antidepressants [14], [48]. This indicates that if an antidepressant-like effect can be blocked by an AMPA antagonist, it is more likely due to the modulation of glutamatergic synapses than the alternative pathways postulated for classic antidepressants. The abolishment of RP's antidepressant-like effect by NBQX in our study thus suggested the role of AMPA receptor activation in RP's antidepressant-like effects.

### Radix Polygalae regulates phosphorylation of the GluR1 AMPA receptor subunit but not mTOR in the hippocampus

**Figure 4:**
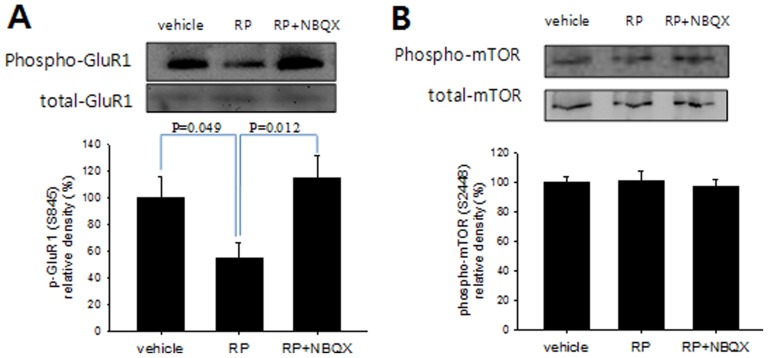
Antidepressant-like effect of Radix Polygalae is associated with phosphorylation of hippocampal AMPA receptor GluR1 but not mTOR. (A) RP treatment (0.1 mg/kg p.o.) reduced hippocampal GluR1 phosphorylation (S845) but not when RP was co-administered with NBQX (10 mg/kg, i.p.) [F(2,15) = 4.38, p = 0.032]. There was no significant change in total GluR1 levels. (B) There was no significant effect on hippocampal phospho-mTOR (S2448) and total mTOR expression by RP or of RP + NBQX. Vehicle: distilled water, RP: Radix Polygalae. All data represent mean ± SEM. ANOVA, Tukey's post hoc test.

The role of AMPA receptors in RP's pharmacological effects was further investigated by checking the status of GluR1, a subunit of the AMPA receptor that exhibits decreased phosphorylation after treatment with ketamine [14] or AMN082, a mGluR modulator that has been shown to decrease behavioral despair in the TST and FST [48] as well as upregulation in PFC synaptoneurosomes following administration of Ro 25-6981, an NR2B receptor subtype specific antagonist with rapid antidepressant effects in the FST and NFST [15]. In our findings, hippocampal GluR1 phospho-serine 845 levels decreased within 30 minutes of RP treatment (0.l mg/kg PO) (Figure 4A), which was consistent with these previous reports. When NBQX was co-administered with RP, however, GluR1 phosphorylation remained unchanged. Since NBQX was shown to block not only the RP-mediated decrease of GluR1 phosphorylation but also its reduction of behavioral despair in the TST, AMPA receptor activation, particularly through the mechanism of GluR1 phosphorylation, seems to be an essential signal regulator in RP-induced antidepressant-like effects.

The mammalian target of rapamycin (mTOR) pathway was another molecular mediator recently implicated in the rapid-onset antidepressant effects of NMDA antagonists [15]. Ketamine rapidly activated this pathway, leading to an increase in the number of synaptic spines in the prefrontal cortex [15]. Additionally, mTOR phosphorylation was increased in peripheral blood cells after ketamine treatment [49], [50]. But mTOR's critical role in mediating rapid-onset antidepressant effect is disputable; according to Autry et al [51], the role of mTOR in ketamine's antidepressant effect may be maintenance rather than rapid induction. According to our results, hippocampal phospho-mTOR levels showed no difference from those in water-administered controls 30 minutes after RP treatment (Figure 3B). But it is still not clear whether phospho-mTOR levels might have changed in the prefrontal cortex or other critical brain regions. However, RP extract rapidly altered GluR1 phosphorylation, which may, in turn, influence the concentrations of membrane-bound AMPA receptors, thus inducing synaptic changes in the hippocampus. Decreased GluR1 phosphorylation at Ser 845 has been associated with removal of AMPA receptors from the cell membrane at the synapse and deemed an integral component of long-term depression [52]. This GluR1 Ser 845 dephosphorylation-dependent removal of AMPA receptors has been shown in response not only to acute bursts of NMDA activity but also high AMPA activation relative to concomitant NMDA activity [53]. The finding of decreased GluR1 phosphorylation at Ser 845 following administration of RP, as well as the abolishment of this effect by AMPA blocker NBQX, is thus consistent with a mechanism in which RP exerts antidepressive effect by antagonizing NMDA, but not AMPA, activity, similarly to that suggested for other rapid-onset antidepressants.

### Glutamatergic synaptic modulation and RP

Various pharmacological reagents acting on glutamatergic synapses can alter the emotional state of a brain. NMDA antagonists, AMPA potentiators, mGluR ligands, and glutamate synaptic release inhibitors, in particular, are suggested as novel candidates for antidepressants that act through glutamatergic pathways. NMDA receptor subtype-specific antagonists like ketamine have been found to produce antidepressant-like effects in clinical and preclinical paradigms [6], [12], [13], [54]. Cyclothiazide, CX-516, and LY392098 showed antidepressant-like effects in association with AMPA receptor potentiation [55], [56]. In addition, Class I mGlu (mGlu1, mGlu5) antagonists, Class II mGlu (mGlu2, mGlu3) antagonists, and Class III mGlu (mGlu4, mGlu6, mGlu7, mGlu8) agonists demonstrated mood-improving effects in animal models [57]. Finally, riluzole (2-amino-6-trifluoromethoxy benzothiazole), an inhibitor of synaptic glutamate release, demonstrated antidepressant effects in a few clinical trials [58], [59].

Among these, NMDA antagonists and mGlu2/3 antagonists were suggested to have rapid-onset antidepressant-like effects in association with the activation of AMPA receptor and mTOR, effects that were blocked when AMPA receptors were occupied by NBQX [60], [61]. In contrast, MTEP (3-[(methyl-1,3-thiazol-4-yl)ethynyl]-pyridine), an mGluR5 antagonist, showed reversals in its antidepressant-like effect when the NMDA but not AMPA receptors were occupied [62]. In our study, the effect of RP in the TST was blocked by pretreatment with the AMPA antagonist NBQX, indicating the possibility of a similar interaction between RP and NMDA the mGlu2/3 receptors.

RP extract contains various substances like tenuigenin, tenuifolin, DISS (3,6′-disinapoyl sucrose), and TMCA (3,4,5-trimethoxycinnamic acid) previously shown to have proliferative and protective effects on hippocampal neurons (tenuigenin) [63], [64], inhibit amyloid-β secretion and improve cognitive function (tenuifolin) [65], [66]exert antidepressant-like effects in chronically stressed rats (DISS) [67], and ameliorate the behavioral effects of cold stress and corticotrophin-releasing hormone administration in rats (TMCA) [68], Among the components of RP, a substance demonstrating either NMDA antagonism or mGluR modulation is highly suggested by both our data and previous work as a key component to the antidepressant effects demonstrated in our experiments.

## Conclusion

The rodent study presented here suggests a rapid-onset antidepressant-like effect of RP in concert with AMPA receptor modulation but not mTOR phosphorylation in the hippocampus. Currently a rush to develop novel antidepressants is ongoing at both the preclinical and clinical stages. RP is a common remedy that is still widely prescribed by practitioners of traditional medicine. RP may possibly substitute for other rapid-onset antidepressants like ketamine that are associated with addiction risk and unacceptable side effects. It will be worthy to further evaluate the pharmacologic effects of RP and especially its constituent parts as candidates for novel antidepressant development.

## Supporting Information

Figure S1 **Chromatogram of tenuifolin (A) and Radix Polygalae (B).** The peak time of tenuifolin was about 13.3 min, and the content comprised 0.006% in RP according to HPLC data.
(TIF)

Figure S2 **The effect of Radix Polygalae on feeding and locomotion.** (A) The effect of RP on locomotor activity. Locomotor activity was measured 30 minutes after a single oral administration of RP (0.1 mg/kg) or distilled water. There were no significant differences in the traveled distance between vehicle and RP fed mice. (B) The effect of RP on feeding amount. Before measurement, mice were fasted but supplied with water for 24 hours. After a single oral administration of RP (0.1 mg/kg) or distilled water, the consumption of chow was measured for 24 hours. No difference emerged between distilled water and RP fed mice. RP: Radix Polygalae. All data represent mean ± SEM.
(TIF)

File S1 **Supporting methods.**
(DOCX)
