# Supplementary figures and images for: Preclinical Evidence of Rapid-Onset Antidepressant-Like Effect in Radix Polygalae Extract
Source: PLoS One. 2014 Feb 10;9(2):e88617. doi: 10.1371/journal.pone.0088617 (PMC3919798; doi:10.1371/journal.pone.0088617)

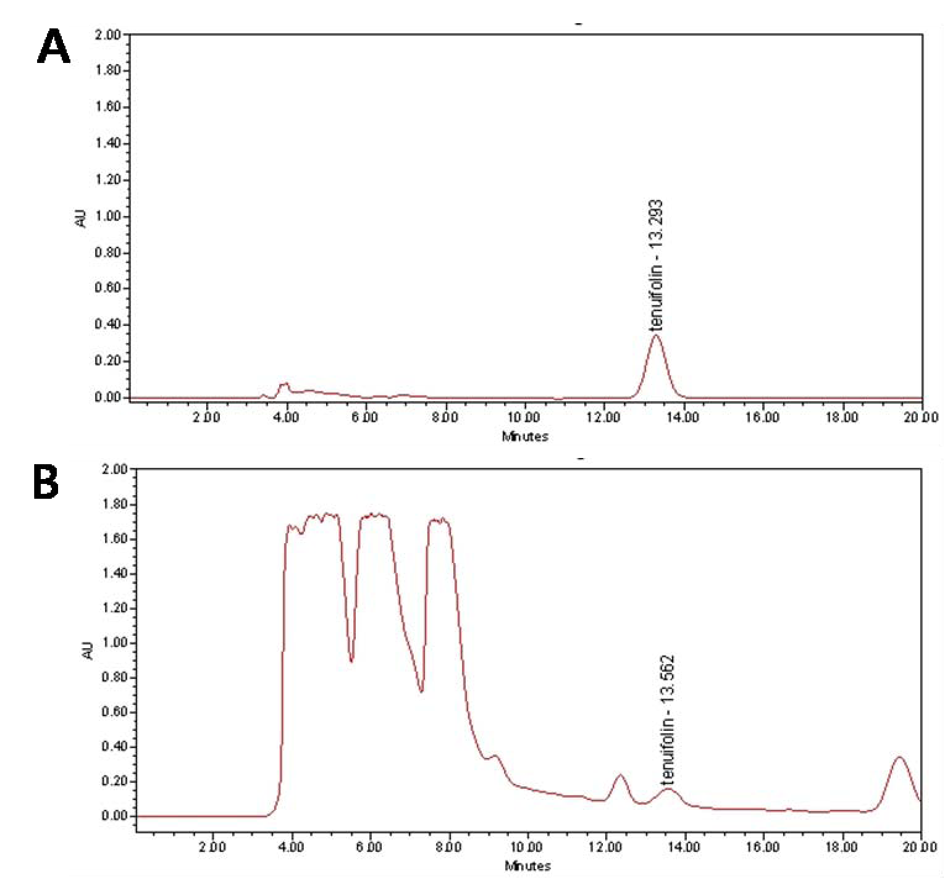

Supplement: Figure S1 — Chromatogram of tenuifolin (A) and Radix Polygalae (B). The peak time of tenuifolin was about 13.3 min, and the content comprised 0.006% in RP according to HPLC data. (TIF) [file pone.0088617.s001.tif]

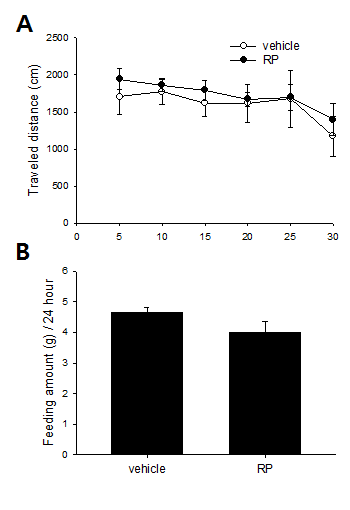

Supplement: Figure S2 — The effect of Radix Polygalae on feeding and locomotion. (A) The effect of RP on locomotor activity. Locomotor activity was measured 30 minutes after a single oral administration of RP (0.1 mg/kg) or distilled water. There were no significant differences in the traveled distance between vehicle and RP fed mice. (B) The effect of RP on feeding amount. Before measurement, mice were fasted but supplied with water for 24 hours. After a single oral administration of RP (0.1 mg/kg) or distilled water, the consumption of chow was measured for 24 hours. No difference emerged between distilled water and RP fed mice. RP: Radix Polygalae. All data represent mean ± SEM. (TIF) [file pone.0088617.s002.tif]
