# Supplementary material for: Preclinical Evidence of Rapid-Onset Antidepressant-Like Effect in Radix Polygalae Extract
Source: PLoS One. 2014 Feb 10;9(2):e88617. doi: 10.1371/journal.pone.0088617 (PMC3919798; doi:10.1371/journal.pone.0088617)
Supplement: File S1 — Supporting methods. (DOCX) [file pone.0088617.s003.docx]

Supporting methods

S1. Identification of tenuifolin in Radix Polygalae by HPLC

S1.1. Apparatus

The HPLC system used consisted of an Alliance 2690 Separation Module, a Waters 996 Photodiode Array Detector operated at 254 nm, and a Millenium^32^ Chromatography Manager Version 3.2. Chromatographic separations were carried out on a Nucleosil C_18_ column (4.0 mm × 250 mm I.D. Waters Corporation, Milford, MA, USA) for tenuifolin at ambient temperature.

S1.2. Standards and reagents

Tenuifolin was purchased from Wako Pure Chemicals Industries, Ltd (Osaka, Japan). Acetonitile (ACN), acetic acid, and phosphoric acid were used in the product of J.T. Baker (Phillipsburg, NJ, USA), Duksan Pure Chemicals Co., Ltd. (Ansan, Kyungkido, Korea), and Yakuri pure chemicals Co., Ltd (Kyoto, Japan). Water used in this assay was ultrapure water.

S1.3. Mobile phase

The mobile phase on tenuifolin consisted of methanol : H_3_PO_4_ = 650: 350 (v/v); the flow rate was 0.7 mL/min. The mobile phase was filtered through a 0.45 μm membrane filter (Millipore, Bedford, MA, USA) and was degassed before use.

S2. Measurement of feeding amount

As appetite change may contribute to the result in the novelty suppressed feeding task, feeding amount was measured after RP treatment. Twenty-four hours before test, mice were individually caged without any chow supplied, but a bottle was left for ad libitum water supply. The next day, the amount of chow consumed was measured for 24 hours after oral administration of RP (0.1 mg/kg, p.o.) or distilled water.

S3. Open field test

As psychomotor stimulation can reduce the immobility in the tail suspension and forced swim tests, locomotor activity was measured after RP treatment. Mice were placed into an open field (50 x 50 x 40 cm) 30 minutes after an oral administration of RP (0.1 mg/kg) or distilled water. The travel distance was measured for 30 minutes using video-tracking software (Smart 3.0, Panlab, Spain).
